# Amalgam

**Published:** 1865

**Authors:** A. T. Johnson

**Affiliations:** Lowell


### AMALGAM.

BY A. T. JOHNSON.

For several years I have read with interest the discussions which have taken place at the conventions of dental associations and elsewhere. Among the various subjects discussed, the use of amalgam or other material than gold, for the filling of teeth, has, at times, occupied a prominent place, finding many able objectors to its use, as well as strenuous advocates in its favor, under certain circumstances and conditions of the teeth.

Many object to its use in toto, saying that any tooth that can be, or is, worth preserving, should be filled with gold, to the exclusion of every other material.

While I deprecate the use of anything but gold for filling front teeth, I would ask what shall we do with unnumbered molars and bicuspids of a very large class of individuals, whose means will not permit them to pay the price of a gold plug, particularly when the cavities are of large size and the charge per plug could not be less than five dollars and upwards, even at the old quotation of gold.

Shall we turn them away, in their inability to pay the price demanded for gold, telling them that it is the only safe and reliable preservation of carious teeth, especially when, in every community, thousands of instances can be cited of teeth that have been filled fifteen or twenty years with other material than gold, and are still in a good state of preservation, which, to say the least, is a tolerable argument in its favor.

Notwithstanding all that has been said against the use of amalgam, about its producing salivation, &c., &c., (which, to my mind, is nonsense of the rankest kind,) it is extensively used by probably a large majority of the profession, a portion of whom, at least, occupy a high rank as practitioners.

The question, then, arises, which of the various campositions that have been offered to the profession for the purpose designated is the best.

Having been in practice between nineteen and twenty years, and, during that period, having made thorough trial of many preparations, I have never as yet found anything that compared favorably with the amalgam prepared by Dr. A. Lawrence, of this city (Lowell).

Having used it for several years, I find it uniform in its results—neither expanding or contracting, producing no discoloration of the teeth, (if properly used,) and, in many cases, no change of color on the surface of the filling.

One little item in passing is noteworthy concerning it, which falsifies the old proverb, "A prophet is not without honor, save in his own country," in the fact that every member of the profession in this city is in the daily use of it.

The demand for it is largely increasing, I learn, which is due, not to puffery or extensive advertising, but to the favor it wins upon trial. From personal knowledge, I know that the inventor and proprietor supervises the preparation of his amalgam, from the incipient stages until it is reduced to powder, in which he is assisted by a small steam engine, which occupies a portion of his laboratory.

Having used this amalgam for several years, (as before said,) with the happiest results, and having adopted a method of using it, particularly in the finishing of the filling that, in some respects, possibly may be original, and thinking, perhaps, that a description of my manner of so doing might not be unacceptable to those of the profession who use, or may use, this amalgam, I will state such method in as brief and explicit language as I possibly can.

After the cavity, or cavities, to be filled are excavated, I put a sufficient quantity of amalgam in a small mortar, add quicksilver, triturate well with pestle, then fill the mortar partially full of alcohol, and then rub the paste with the pestle thoroughly. If the alcohol is discolored by so doing, turn it off and put in more, and so on, until no discoloration is apparent. Then put the paste in a bit of linen or cotton cloth, press out the superfluous quicksilver, *rub dry*, and it is ready for use. In filling crown cavities, I pack them more than full, wait a short time for it to harden, in the meantime turning a few drops of a preparation called "sozodont" (which acts as a lubricator) into a tumbler of soft water, shaking the same. I then take a small pellet of fine cotton in tweezers—wet the same in contents of tumbler—touch it to powdered pumice stone, and pass it lightly back and forth across the filling. I do this at intervals (using more strength as it hardens) several times, sometimes with pumice and sometimes without, and the result is a beautiful filling, flush with and about as smooth as the enamel, for which a burnisher is a needless instrument. For approximal and buccal cavities, I use for packing the filling an instrument similar in shape to the flat surface of a burnisher, but much thiner, the end and edges being sharp enough to shave off the surplus material. I then wet the end of a thin wedge shaped stick, touch it to the pumice, and pass it gently between the teeth; after which I take a thin wisp of cotton, twisted slightly, or bit of narrow tape, wet the same, add pumice, and pass it over the surface of the filling. I do this at intervals, (as before mentioned,) and the final result is a filling alike satisfactory to operator and patient. It requires more time than in the usual manner of finishing such fillings with a burnisher; but, in my estimation, it is well spent, most certainly as far as the patient is concerned, as well as satisfaction to the operator, in the pleasing results of his manipulations.

Trusting that I may have uttered no treason against the art preservative of the masticating organs in the above communication, I offer it to the consideration of your readers, if you should deem it worthy a place in the pages of your journal, hoping that a perusal of the same may, at least, be productive of no harm.

*Lowell, March*, 1865.

